# Cold-inducible RNA binding protein alleviates iron overload-induced neural ferroptosis under perinatal hypoxia insult

**DOI:** 10.1038/s41418-024-01265-x

**Published:** 2024-02-22

**Authors:** Xiaozheng Zhu, Ruili Guan, Yuankang Zou, Ming Li, Jingyuan Chen, Jianbin Zhang, Wenjing Luo

**Affiliations:** Department of Occupational and Environmental Health and the Ministry of Education Key Lab of Hazard Assessment and Control in Special Operational Environment, School of Public Health, Fourth Military Medical University, Xi’an, China

**Keywords:** Neurological disorders, Neural ageing

## Abstract

Cold-inducible RNA binding protein (CIRBP), a stress response protein, protects cells from mild hypothermia or hypoxia by stabilizing specific mRNAs and promoting their translation. Neurons subjected to hypobaric hypoxia insult trigger various cell death programs. One of these is ferroptosis, a novel non-apoptotic form of programmed cell death, which is characterized by excessive iron ion accumulation and lipid peroxidation. Here, we establish that CIRBP can regulate neuronal ferroptosis both in vivo and in vitro. We observe that hypoxia leads to neuronal death via intracellular ferrous iron overload and impaired antioxidant systems, accompanied by suppressed CIRBP expression. Genetic enrichment of CIRBP in hippocampal neurons CIRBP^Tg^ mice bred with Emx1-Cre mice attenuates hypoxia-induced cognitive deficits and neuronal degeneration. Mechanistically, CIRBP alleviates neuronal ferroptosis and intracellular ferrous ion accumulation by binding to the mitochondrial ferritin (FTMT) 3’UTR to stabilize mRNA and promote its translation. Our novel study shows the critical role of CIRBP in the progression of ferroptosis, and provides promising therapeutic target for hypoxia-induced neurological diseases.

## Introduction

Due to the demand for employment and the continuous optimization of transportation options, the number of migrants who live at high altitudes is continuously rising. According to the Lancet, more than 140 million people were residing in high-altitude regions above 2500 meters as early as 2001 [1]. The drop in barometric pressure leads to a decrease in ambient oxygen partial pressure throughout the oxygen transport chain, which in turn leads to a range of altitude-related illnesses [2, 3]. The incidence of miscarriage, premature birth, and fetal respiratory distress are also significantly higher in the pregnant population in high-altitude areas than lower plain areas [4–6]. Early life is a crucial stage of neurodevelopment, and the damage is often irreversible and predisposes to cognitive, affective, depressive, and other brain function disorders. Hypoxia exposure in early life can trigger neuronal deficits, damage the synaptic structure, and arrest the formation of neuromyelin. According to the Lancet, nearly half of postnatal infants who suffered from intrauterine hypoxia would result in health sequelae with abnormal cognitive development [7]. In conclusion, hypoxia contributes to severe impairment in cognitive function.

Damage to neurons is largely irreversible because nerve cells are highly differentiated and no longer have the capacity to proliferate. Ferroptosis is a novel non-apoptotic form of programmed cell death that depends on the ferrous ions overload in cells and differs morphologically and genetically from apoptosis, necrosis, and autophagy [8, 9]. The primary mechanism of ferroptosis is the catalysis of lipid peroxidation by lipoxygenase or ferrous ions, which results in the highly expressed polyunsaturated fatty acids on cell membranes, followed by cell death [10]. Additionally, an imbalance of intracellular redox status and a depressed expression of the antioxidant enzyme GPX4 occur [11]. Regarding morphology, ferroptosis results in shrunken mitochondria, fewer mitochondrial cristae, and minimally altered nuclear morphology [12]. According to various reports, in 1% oxygen environment, hypoxia leads to enhanced ROS production in mitochondrial complex III, which directly promotes lipid peroxidation by damaging the mitochondrial membrane [9]. In addition, elevated levels of cellular hydrogen peroxide can support the Fenton reaction, thereby further promoting ferroptosis by depleting cellular antioxidant capacity. However, it is still unknown whether maternal hypoxia can cause neuronal ferroptosis in the fetus.

Cold-inducible RNA-binding Protein (CIRBP), also known as A18 hnRNP, is found after cells are subjected to toxic cold shock [13]. By maintaining specific mRNA stability and promoting their translation, CIRBP can promote cells proliferation and migration [14]. Our previous study showed that CIRBP has neuroprotective effects and improves learning memory function by promoting the proliferation of neural stem cells under hypoxic stimulation [15]. In addition, persistent hypoxia exposure induced hypermethylation of the CIRBP promoter region to suppress its expression, accompanied by a considerable drop in coenzyme Q10 (CoQ10) concentration and an imbalance in redox response [16]. Furthermore, emerging evidence showed that CIRBP alleviates amyloid β (Aβ)-induced neurotoxicity by inhibiting pro-apoptotic gene expression and ROS production [17]. Nevertheless, the expression of CIRBP and its precise role in ferroptosis under perinatal hypoxia condition have not been studied.

In the present study, we developed a model of pregnant mice exposed to hypoxia. We established that there were symptoms of ferroptosis and poor cognitive performance in the offspring. As seen in the suppression of CIRBP expression at late-stage exposure, we generated neuron-specific enhancement CIRBP transgenic mice by crossing CIRBP^Tg^ mice with Emx1-Cre mice, and found that overexpression of CIRBP in the hippocampus partially attenuated the ferroptosis and memory impairments in hypoxia models. Mechanistically, CIRBP directly binds to mitochondrial ferritin (FTMT) mRNA 3’UTR region to maintain the stability and inhibit iron accumulation, therefore alleviating ferroptosis.

## Materials and methods

### Animals and hypoxia insult

CIRBP transgenic (CIRBP^Tg^) mice were designed and developed by Biocytogen Co. Ltd (Beijing, China) and transferred from the C57BL/6 (C57) background. Emx1-Cre mice were purchased from the Jackson Laboratory (Bar USA, Stock #005628) and bred in the Experimental Animal Central of Fourth Military Medical University. Emx1-Cre mice were mated with homozygous CIRBP^Tg^ mice to generate conditional CIRBP enhancement mice (CIRBP^Tg: Emx1-Cre^). Littermates lacking the CRE gene (CIRBP^Tg^) were used as controls. All strains were maintained on a C57 background. Genotyping was validated as previously described [18], and the primer sequences used for gene identification are shown in Supplementary Tables.

All mice were kept in the specific pathogen-free (SPF) environment for at least one week, accompanied by free diet and water, 12-h light/dark cycle. All animal procedures were conducted in accordance with the Guidelines for the Care and Use of Laboratory Animals in China, and approved by the experimental animal ethics committee of Fourth Military Medical University.

The presence vaginal plug date was defined as embryonic day 0.5 (E0.5), and birth was designated as P0. On postnatal day 7, when ferroptosis had not yet occurred, both normoxic and hypoxic littermates were injected intraperitoneally with an ferroptosis inhibitor liproxstatin-1 (Lip-1, 10 mg/kg) every other day until P21. For perinatal hypoxia insult, pregnant mice at E10.5 were randomly assigned to decompression chamber (Fenglei Co. Ltd., China) until the pups were removed on the designated date after birth. Hypoxic chamber parameters were set to 4000 m altitude (equal to partial 12.7% O_2_ pressure) as described previously [19].

### Cell culture and hypoxic exposure

Primary cortical neurons were cultured as previously described. E16.5 C57 mice embryos were placed in pre-cooled HBSS Hanks. The brain tissues were cut into pieces, digested with 0.25% trypsin, and terminated with FBS. After filtering the tissue fragments with a strainer, the resultant solution containing cells was centrifuged and cells were seeded in Neurobasal/B27 medium supplemented with 1.25 μmol/L L-glutamine and 1% penicillin/streptomycin. Half of the medium was replaced every 3 days, and neurons were used for experiments after the second medium change.

For hypoxic exposure, HT22 and SH-SY5Y cell lines and primary cortical neurons were incubated in a hypoxic chamber with 1% O_2_, 5% CO_2_, balanced by N_2_ (DWS-H85, Don Whitley, UK). Culture medium needed to be acclimated 12 hours in advance.

### Lentiviral transfection

CIRBP mRNA sequence was obtained from NCBI and listed in Supplementary tables. Lentiviruses carrying CIRBP (pLenti-CMV-Cirbp-P2A-EBFP2-3Flag-WPRE) or FTMT (pcSLenti-EF1-EGFP-CMV-FTMT-3xFLAG-WPRE) were obtained commercially from Obio Technology, China.

The lentivirus-based short hairpin RNA (shRNA) targeting CIRBP, pSLenti-U6-shCirbp-CMV-EGFP-F2A-Puro-WPRE, was purchased from Obio Technology, China. The target sequence was as follows: 5′-GGGTGGCAGCTATGGTTAT-3′.

For stable lentiviral transfection in HT22 cell line and primary neurons, cells were grown in 6-cm dishes at a density of 5 × 10^5^, and lentivirus was added to the medium at a MOI of 20 or 60, respectively. The following day, the medium was exchanged. qPCR was used to confirm the impact of gene overexpression or interference.

### Morris water maze test

The ability of spatial learning and memory in mice were evaluated by morris water maze test following a previous protocol with a slight modification [20]. Briefly, at the end of hypoxia exposure, mice received a consecutive 5-day maze training with 4 trials per day to search for the hidden platform prior to test. On the test day (Day 6), the probe trial was conducted by removing the platform and each mouse was given 120 s to seek the platform. The latency and frequency to platform and duration in the target quadrant were recorded.

### Novel object recognition test

The novel object recognition test was performed as previously described [21]. Prior to training, the mice were placed in the room for 30 minutes to adapt to the novel environment. Then mice were left in an open field experimental device (40 cm × 40 cm × 40 cm) with two similar objects spaced equally distant from the edge for 5 min. One hour later, the animals were put back in the apparatus with one familiar and one novel object. The recognition index reflected the percentage of time spent on novel object.

### Open field test

The open field test can used to identify locomotor activity and exploratory behavior in mice. Briefly, the mice were put gently in one corner of the testing chamber and given 5 minutes of unrestricted mobility, which was tracked by an automated video tracking system. The time and distance spent in central area were recorded and analyzed.

### Assessment of neuronal viability

Cell viability was assessed with a CCK-8 assay and PI staining (Yeasen, China). After treatment, cells were washed with PBS, and CCK-8 reagent was added to the culture medium 2 hours before analysis. Following the incubation, the absorbance at 450 nm was detected with a microplate reader. For PI staining, a PI staining kit was used according to the manufacturer’s instructions. In brief, cells were incubated with 5 μg/mL PI for 15 minutes. Then, the medium was discarded. After fixation for 15 minutes using 4% paraformaldehyde, cells were stained with DAPI. Double positive (PI+/DAPI+) was considered as dead cells.

### Immunofluorescence staining

Mice were euthanized, and brains were removed and fixed in 4% paraformaldehyde, dehydrated in 30% sucrose in PB, and embedded in OCT. The brain tissue was subsequently sliced into 14 μm-thick slices. For immunofluorescence staining, briefly, the sections were permeabilized with Triton 0.3%, blocked with BSA 5%, and incubated with a primary antibody overnight. After rinsing, slices were incubated with fluorescent-conjugate secondary antibody and mounted with mounting media containing DAPI. For TUNEL staining, the procedure was carried out in accordance with the TUNEL staining kits instructions (Servicebio, China). Images were acquired with ZEISS Axiovert 200 fluorescent microscope and analyzed with ImageJ. Additional antibody details are shown in the Supplementary Tables.

### Immunoblotting analysis

Total proteins from hippocampus or cells were extracted with RIPA lysis buffer (Beyotime Biotechnology, China). 40 μg of protein was electrophoresed on SDS-PAGE and transferred onto a PVDF membrane (Merck, Germany). Following blocked in 5% skim milk for 1 hour at room temperature, the membrane was incubated overnight at 4 °C with primary antibodies. After three TBST washes, the membrane was incubated for 1 hour with HRP-conjugated secondary antibodies and visualized with ECL reagents (Millipore, USA). Additional antibody details are shown in the Supplementary Tables.

### Confocal microscopy imaging

A confocal laser scanning microscope (Leica, Germany) with a 60× or 100× oil immersion objective lens was used to view the fluorophore-labeled cells. Primary neurons and HT22 cells were seeded in confocal dishes at a density of 2 × 10^5^, and cultured at 37 °C before treatment.

The BioTracker FerroOrange Live Cell Dye (Merck, Germany) is an orange fluorescent probe that detects exclusively labile iron (II) ions (Fe^2+^). According to the manufacturer’s instructions, cells pretreated with hypoxia were incubated with 1 μM FerroOrange probe at 37 °C for 30 min, followed by a PBS wash through and the addition of 2 mL HBSS on confocal microscopy for photography. The fluorescence of Fe^2+^was examined at excitation wavelength of 561 nm.

To assess lipid peroxidation, BODIPY-C11 lipid peroxidation sensor (Thermo Fisher Scientific, USA) was conducted in accordance with manufacturer’s protocol. Primary neuronal and HT22 cell lines were exposed to hypoxia for 9 days and 48 hours, respectively, and then 2 μM BODIPY 581/591 was added and incubated for another 30 minutes. Cells were rinsed twice with PBS and subjected to live cell imaging at 488 and 565 nm excitation wavelengths to visualize the oxidized and non-oxidized substrates, respectively.

### Flow cytometry

Cellular mortality and BODIPY-C11 lipid peroxidation were evaluated by FACS caliber flow cytometry analysis (Beckman Coulter, USA) according to manufacturer’s instructions. PI (50 μg/mL) was used to detected cell mortality, BODIPY-C11 (2 μM) was applied to detect intracellular lipid peroxidation. Briefly, cells were trypsinized, washed with PBS three times, and stained with PI dyes or BODIPY-C11 for 20 min at 37 °C. Then cells were counted with flow cytometry and analyzed using Flowjo software.

### Transcriptome RNA-seq analysis and qPCR

Transcriptome analysis of CIRBP knockdown and scrambled control HT22 cells was conducted using RNA sequencing (RNA-seq) as described previously [22]. Total RNA was isolated using the Trizol Reagent (Invitrogen, USA), followed by the detection of RNA integrity and quantity. mRNA was purified from total RNA using poly-T oligo-attached magnetic beads and randomly interrupted into short fragments by divalent cations. The cleaved RNA fragments were then reverse-transcribed to construct the final complementary DNA (cDNA) library, which was then sequenced on DNBSEQ-T7 with PE150 model at GeneCreate Biological Engineering (Wuhan, China).

Total RNA samples were extracted from hippocampus tissue homogenates and cells using the RNAiso plus reagent (Takara, Japan) according to the manufacturer’s procedure. Extracted RNA was converted to cDNA using a reverse transcription kit. Gene expression was quantified using qPCR and the SYBR Green PCR Master Mix (Cwbiotech, China) standard procedure. The relative expression levels were determined relative to actin using the 2^-ΔΔCt^ method. The primer sequences are described in the Supplementary material.

### Transmission electron microscopy

Following anesthesia, mice were perfused with normal saline and 4% paraformaldehyde solution, the hippocampus was removed and trimmed to 1 mm^3^ and treated with 4% glutaraldehyde overnight. Subsequently, the dissected hippocampus was rinsed 3 times with PBS and post-fixed with 1% osmium solution. This was followed by an additional rinse and dehydration with gradient acetone, staining with 2% uranyl acetate, and embedding with Epon. Finally, sections were stained with uranyl acetate and lead citrate and photographed by transmission electron microscopy (Hitachi, Japan).

### Measurement of iron indices

Iron Assay Kit (Abcam, UK) was performed to detect tissue nonheme iron according to the instructions. Briefly, tissue was minced and homogenized. The supernatant after centrifugation was applied to the probe and incubated for 60 minutes. Absorbance signals were detected with microplate reader and the specific parameters were calculated using a standard curve.

### Measurement of GSH and MDA levels

Intracellular GSH and GSSG level was measured by GSH/GSSG assay kit (Beyotime, China) according to the manufacturer’s instruction. Tissue homogenates and cells were lysed and the supernatant was centrifuged for further detection of intracellular GSH and GSSG.

The MDA assay was performed according to the manufacturer’s instructions using the Lipid Peroxidation MDA Assay Kit (Beyotime, China). Samples and standards OD values were analyzed at 532 nm. MDA concentrations were quantified as μmol/g protein.

### GPx activity assays

The activity of glutathione peroxidase (GPx) was evaluated by colorimetric assay (Abcam, UK). Briefly, samples were taken and depleted of all GSSG by incubating them for 15 minutes with glutathione reductase (GR) and reduced glutathione (GSH). GPx activity was evaluated by incubation for 10 minutes with hydroperoxide and detected absorbance at 340 nm.

### CHX chase assay and analysis of mRNA stability

We investigated the influence of CIRBP on the stability of FTMT through the cycloheximide (CHX) experiment [23]. Stable interference HT22 cells were treated with 5 μg/mL CHX for 0, 3, 6, 9 hours to block translation, and cell lysates were electrophoresed by immunoblotting.

mRNA stability was conducted by actinomycin D assay [24]. HT22 cells were seeded in 6-well plates and treated with 10 μg/mL actinomycin D for 0, 1, 2, 4, 8, and 12 h to block transcription. We used Trizol for RNA extraction and detected the level of FTMT mRNA remaining.

### Dual-luciferase reporter activity assays

The murine FTMT 3′UTR or FTMT-NC fragments were cloned into pMIR-Report luciferase plasmid (Obio Technology, China). For the luciferase reporter assays, stable CIRBP overexpression or interference HT22 cells were transfected with luciferase plasmid containing FTMT 3’UTR and CMV reference plasmid. The luciferase activity was measured with the Dual-Luciferase Reporter Assay System (Promega, USA) according to the manufacturer’s instructions.

### RNA immunoprecipitation

The RNA immunoprecipitation (RIP) assay was conducted using RIP kit (BersinBio, China) in accordance with the manufacturer’s instructions. In brief, equal amounts of cell lysates were incubated overnight with CIRBP antibodies or IgG. Mixed solutions collected by magnetic beads were rinsed 3 times, and the RNA samples were extracted with Trizol reagent and detected by qPCR.

### Statistical analysis

All the assays were repeated three times independently, and data was expressed as the Mean ± SEM. The statistical analysis was carried out using the SPSS 20.0 software. Two-tailed unpaired Student’s t-test and multi-way ANOVA were utilized to determine differences between treatment and control groups. Differences were considered statistically significant when *P* < 0.05.

## Results

### Abnormal development and cognitive impairment in offspring induced by perinatal hypoxia exposure

**Fig. 1:**
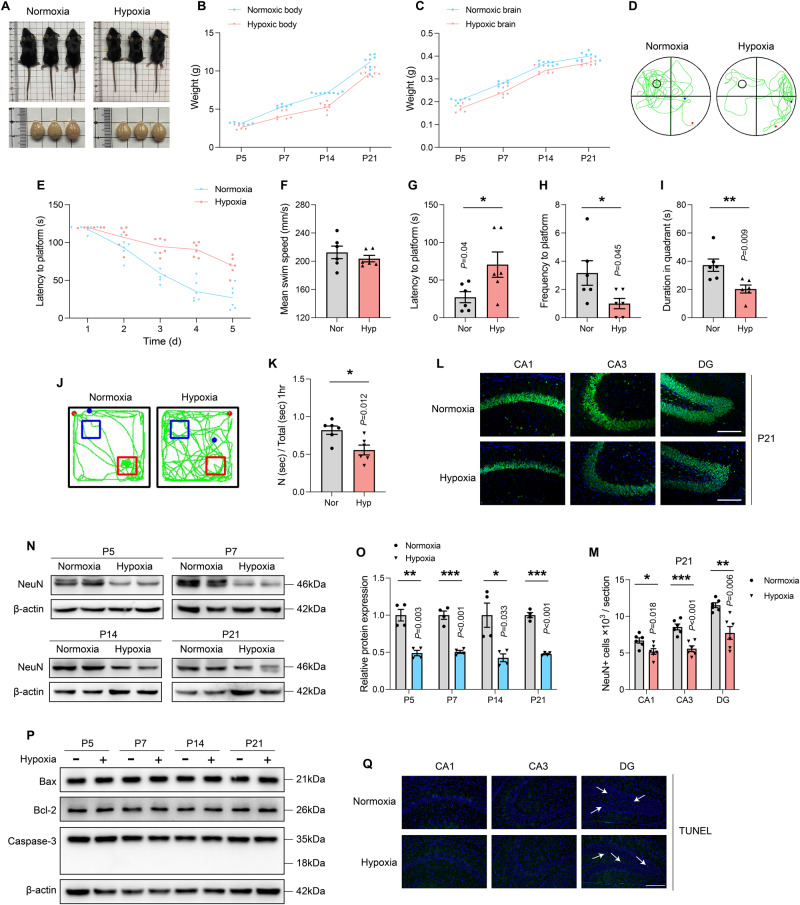
Exposure to perinatal hypoxia causes abnormal development and cognitive damage in offspring. **A** The representative images of the mice and the whole brain under normoxia or hypoxia conditions on postnatal day 21. **B** The body weights of mice in perinatal hypoxia insult and age-matched controls on P5, P7, P14, and P21. **C** The brain weights of mice under normoxia or hypoxia on the designated date after birth. **D** The representative swimming tracks of mice on P21 in the Morris water maze (*n* = 6). **E** The latency to the platform during the training sessions. **F** The mean swim speed, **G** the latency to a hidden platform, **H** the frequency to a hidden platform, as well as **I** the time in the target quadrant on the test day in the Morris water maze (*n* = 6). **J** The representative travel tracks of mice on P21 in the novel object recognition test (*n* = 6). **K** The discrimination index for mice in the novel object recognition test (*n* = 6). **L** The representative images of immunostaining (green for NeuN, blue for DAPI) in the CA1, CA3, DG regions of hippocampus under hypoxia condition. Scale bars is 200 μm. **M** Counts of NeuN+ neurons in CA1, CA3 and DG regions of P21 mice. **N** Hippocampus tissues were homogenated and lysed and protein samples were prepared. Expression levels of NeuN protein was tested by immunoblotting. **O** Intensity quantification was performed by Image J and normalized to β-actin. (*n* = 4). **P** Protein samples of hippocampus tissues were collected and apoptosis-related proteins were detected by immunoblotting. Represented bands for Bax, Bcl-2, Caspase-3 in hippocampus. **Q** The representative images of TUNEL staining (green for TUNEL positive, blue for DAPI) in the CA1, CA3, DG regions of hippocampus under hypoxia condition. Data were showed as mean ± standard error of mean (SEM) of at least three independent experiments. Statistical analyses were carried out using a two-tailed unpaired Student’s *t* test for comparations between two groups. *P ≤ 0.05, **P ≤ 0.01, ***P ≤ 0.001. Source data are provided as a Supplemental Material file.

Physiological indicators (Fig. 1A) in the hypoxic and normoxic groups on the 5th, 7th, 14th, and 21st day after birth addressed the impact of hypoxia during pregnancy on offspring developmental functions. Results revealed that the hypoxic group body weight, brain weight, and hippocampus weight were significantly lower than those of the normoxic group (Fig. 1B, C; SFig. 1A, B; and STable 6 and 7), indicating that hypoxia leads to aberrant pup development. Next, we performed functional tests on learning, memory, and spatial exploration in hypoxia-treated pups, noting no significant differences in basic motor skills between them and the normoxic group (Fig. 1F and SFig. 1D). In Morris water maze tests, we discovered that hypoxic pups showed significantly poorer learning performance during the training sessions (Fig. 1E; STable 8). In the probe trial, mice in the hypoxic group exhibited a longer latency time to reach the hidden platform, lesser frequency of crossing platform and a shorter duration in the target quadrant (Fig. 1D, G–I). Additionally, results of the novel object recognition test (Fig. 1J, K), which was performed to examine short-term memory retention, showed that hypoxia-exposed pups spent less time on the novel object, indicating short-term memory impairment due to hypoxia insult. Furthermore, open-field experiments showed that hypoxia exposure led to reduced frequence, shorter times, and fewer distances traveled in the central area (SFig. 1C, E–G). Taken together, these findings establish that perinatal exposure to hypoxia impairs offspring learning, memory, and spatial exploration.

Hippocampal neurons in the CA1, CA3, and DG regions are crucial for cognitive learning [25]. Thus, we explored whether hypoxia affected the number of hippocampal neurons on P5, P7, P14, and P21. Immunofluorescence results revealed a decline in the number of neurons throughout the subregions of the hippocampus at various time periods, except for the hippocampal CA1 region in the P5 group (Fig. 1L, M and SFig. 1H–M). Consistently, the expression of NeuN in hippocampus was significantly decreased at all specified times (Fig. 1N, O). To explore the key contributors to the decreased number of neurons in the hippocampus, we examined whether neuronal apoptosis occurred during perinatal hypoxic exposure. The results showed that, regrettably, the apoptosis-related proteins (Bax/Bcl-2, cleaved caspase-3) were not significantly altered (Fig. 1P and SFig. 1N), and TUNEL staining likewise revealed no difference in the number of TUNEL-positive cells in the hippocampus of the normoxic and hypoxic groups (Fig. 1Q and SFig. 1O). These findings demonstrate that hypoxia resulted in hippocampal neuronal death rather than apoptosis, a substrate for the behavioral data shown above.

### Hypoxia-exposed neurons in the hippocampus develop molecular and morphological features of ferroptosis

**Fig. 2:**
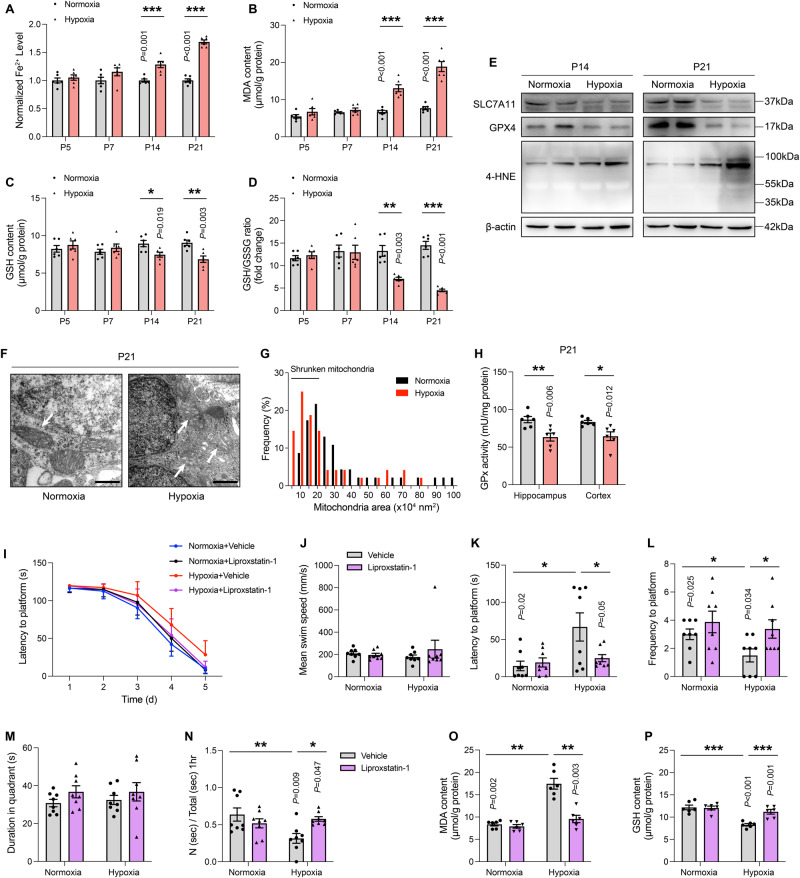
Hypoxia-exposed neurons in the hippocampus develop molecular and morphological features of ferroptosis. **A** The tissue iron content in the hippocampus exposed by hypoxia on P5, P7, P14, and P21, data were normalized to normoxia (*n* = 6). **B** The MDA content (μmol/g protein) in the hippocampus of mice under hypoxia conditions (*n* = 6). **C** The GSH content and **D** the GSH/GSSG ratio in the hippocampus of mice with or without hypoxia insult on the designated date after birth (n = 6). **E** Protein samples of P14 and P21 hippocampus were collected and ferroptosis-related proteins were detected by immunoblotting. Represented bands for SLC7A11, GPX4, 4-HNE in hippocampus. **F** Transmission electron microscopy pictures show shrunken mitochondria of P21 hippocampal neurons under hypoxia conditions. Scale bars is 400 nm. **G** Frequency of mitochondrial area size around the nucleus demonstrated. The number of mitochondria is counted as 50, approximately. **H** The GPx activity (mU/mg protein) in the hippocampus and cortex of P21 pups (*n* = 6). **I** The latency to the platform during the training sessions. **J** The mean swim speed, **K** the latency to a hidden platform, **L** the frequency to a hidden platform, as well as **M** the time in the target quadrant on the test day in the Morris water maze (*n* = 8). **N** The discrimination index for mice in the novel object recognition test (*n* = 8). **O** The MDA content (μmol/g protein) in the hippocampus of P21 mice under hypoxia conditions with or without Lip-1 administration (*n* = 8). **P** The GSH content in the hippocampus of P21 mice with or without intraperitoneal Lip-1 (*n* = 8). Data were showed as mean ± standard error of mean (SEM) of at least three independent experiments. Statistical analyses were carried out using a two-tailed unpaired Student’s *t* test for comparations between two groups. *P ≤ 0.05, **P ≤ 0.01, ***P ≤ 0.001. Source data are provided as a Supplemental Material file.

Ferroptosis, a recently described modality of programmed cell death, is characterized by the overload of ferrous ions and closely associated with hypoxia [26]. Firstly, we assessed the content of ferrous ions in the hippocampus at various times, noting that the iron levels in the P14 and P21 hypoxic pups were significantly increased (Fig. 2A). Consistent with iron accumulation, MDA content, one of the products of lipid peroxidation, was significantly upregulated on P14 and P21 (Fig. 2B), accompanied by decreased GSH content and GSH/GSSG ratio (Fig. 2C, D). To further confirm that ferroptosis triggered neuronal death, we analyzed the expression of specific ferroptosis-associated genes in the hypoxic pups, including solute carrier family 7 member 11 (SLC7A11), GPX4, and 4-hydroxynonenal (4-HNE). Among those genes, GPX4, which can effectively inhibit ferroptosis, was upregulated during early exposure and then significantly suppressed from P14 to P21. SLC7A11, a cofactor of GPX4 which increases GSH production, was downregulated only on P21. 4-HNE, another lipid peroxidation product, was also elevated only at latter stage of exposure (Fig. 2E and SFig. 2A–C, E–G). Additionally, hypoxia exposure altered GPx activity both in the cortex and hippocampus of P21 pups, while there was no difference on P14 (Fig. 2H and SFig. 2H). In the perinuclear and cytoplasm of hippocampal neurons of the hypoxic group, we also uncovered smaller, ruptured mitochondria, and reduced mitochondrial cristae, all of which are hallmarks of ferroptosis [27], (Fig. 2F and SFig. 2I). Statistically, the quantitative data illustrated that the frequency of shrunken mitochondria was significantly increased in the P14 and P21 groups (Fig. 2G and SFig. 2J). Combined, these data indicate that ferroptosis mediates the hypoxia-induced neuronal loss.

To validate whether perinatal hypoxia was vulnerable to neuronal ferroptosis in general, neonatal littermates (P7) were intraperitoneally injected with ferroptosis inhibitor liproxstatin-1 (Lip-1, 10 mg/kg) every other day. Morris water maze results showed administration of Lip-1 reversed the spatial learning and memory ability of littermates which were sustained exposed to hypoxia until postnatal day 21 (Fig. 2I–M and STable 9). In addition, the administration of Lip-1 significantly improved the short-term memory capacity and spatial exploration characteristics of the hypoxic group of litters, as judged by the novel object recognition test and the open field experiment, respectively (Fig. 2N and SFig. 2K–N). We then investigated the effects of Lip-1 on ferroptosis in hippocampal neurons. As expected, Lip-1 administration effectively ameliorated MDA accumulation (Fig. 2O) and elevated GSH levels (Fig. 2P and SFig. 2D). Taken together, these data show that ferroptosis is one of the factors contributing to neuronal loss secondary to hypoxic exposure.

### Ferroptosis inhibitors ameliorates the neuronal death induced by hypoxia

**Fig. 3:**
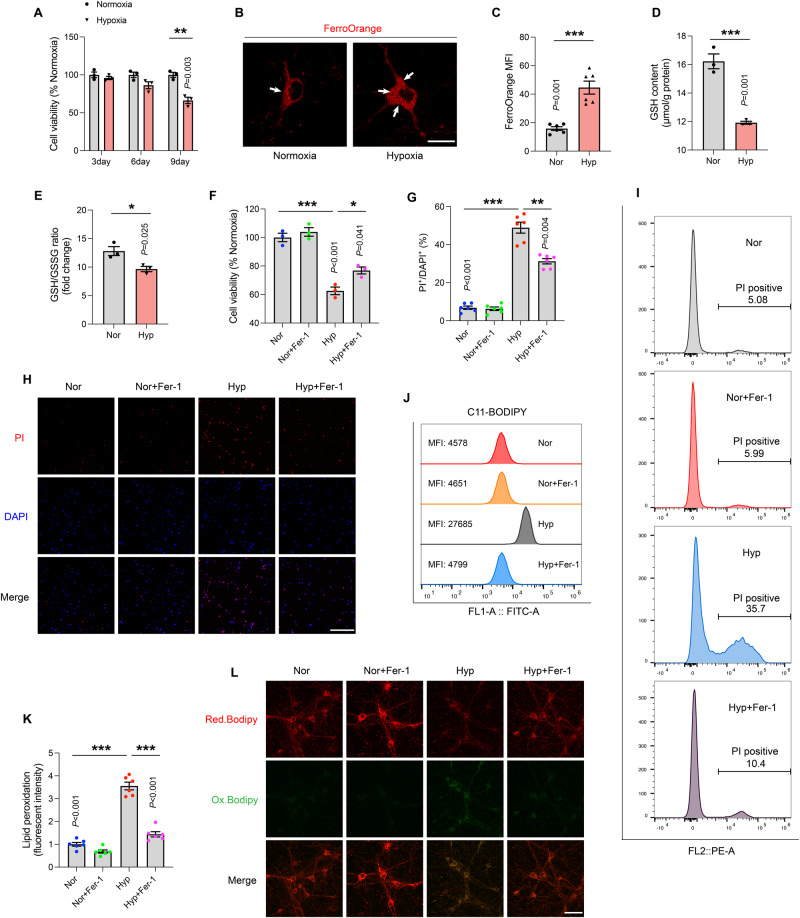
Ferroptosis inhibitors ameliorates the neuronal death induced by hypoxia in vitro. **A** Primary neurons were exposed to 1% O_2_ for 3, 6, and 9 days. The cell viability was accessed by CCK8 assays (*n* = 3). **B** The content of intracellular ferrous ions exposed to hypoxia for 9 days was detected by FerroOrange probe. Scale bar is 20 μm. **C** The iron levels are determined based on the intensities measured by ImageJ (*n* = 6). **D** The GSH content and **E** the GSH/GSSG ratio in the primary neurons under hypoxia exposure. **F** The cell viability exposed by hypoxia with or without Fer-1 (1 μmol/L) administration (*n* = 3). **G** Quantitative analysis of the number of double-positive cells as a proportion of total cells (*n* = 6). **H** PI staining was utilized in primary neurons viability assessment (red for PI, blue for DAPI), PI/DAPI dual-positive cells were considered to dead cells. Scale bar is 200 μm. **I** Cell death was measured by flow cytometry labeled PI staining. **J** The levels of C11-BODIPY in each group were determined by FACS analysis. **K** Quantitative analysis for primary neurons lipid peroxidation (the ratio of green fluorescence to red fluorescence) under hypoxia or Fer-1 administration (*n* = 6). **L** The representative pictures of lipid peroxidation assay (red for reduction, green for oxidation). Scale bar is 50 μm. Data were showed as mean ± standard error of mean (SEM) of at least three independent experiments. Statistical analyses were carried out using two-way ANOVA and multiple *t* test. *P ≤ 0.05, **P ≤ 0.01, ***P ≤ 0.001. Source data are provided as a Supplemental Material file.

To further confirm that ferroptosis is responsible for hypoxia-induced neuronal death, primary cortical neurons and the neuronal cell lines (HT22 and SH-SY5Y cell line) were subjected to 1% hypoxic condition. CCK-8 and PI staining were used to assess cell viability. The activity of primary neurons significantly decreased after 9 days of 1% O_2_ exposure (Fig. 3A). In contrast, HT22 cell viability decreased after 24 hours of hypoxic exposure, with most pronounced significant difference observed at 48 hours (SFig. 3A). Ferrous ion levels in living cells were measured using the FerroOrange probe, noting that its content in primary neurons and HT22 cells in the hypoxic group was significantly upregulated (Fig. 3B, C and SFig. 3B, C). Hypoxic exposure also caused a significantly down-regulation in intracellular GSH levels and GSH/GSSSG ratio, consistent with the in vivo animal results (Fig. 3D, E and SFig. 3D, E). To determine whether ferroptosis mediated hypoxia-induced neuronal deficits, we treated the cells with a ferroptosis inhibitor, Fer-1 (1 μmol/L). Fer-1 attenuated the effects of hypoxia-induced cell death both in primary neurons, HT22 cells and SH-SY5Y cells (Fig. 3F and SFig. 3F, G). The number of PI-positive cells significantly increased after hypoxic insult, and treatment with Fer-1 partially diminished this effect (Fig. 3G, H and SFig. 3I, K). Flow cytometry results similarly corroborated the above results (Fig. 3I).

Dyshomeostasis in cellular redox and the ensuing cellular lipid peroxidation are hallmarks of ferroptosis. BODIPY 581/591 C11 has been profitably used to detect lipid peroxidation in living cells [28]. When lipid hydroperoxide oxidizes cells, the fluorescence emission peak shifts from 590 nm (red) to 510 nm (green). Hypoxic exposure resulted in significant lipid peroxidation in primary neurons and HT22 cells, as seen in Fig. 3K, L and SFig. 3J, L, with increased ratio of green to red fluorescence. In contrast, most normoxic cells exhibited homogeneous red fluorescence. Meanwhile, the levels of lipid peroxidation detected by FACS were effectively modulated by Fer-1 both in HT22 cells and SH-SY5Y cells (Fig. 3J and SFig. 3H). Therefore, treatment with Fer-1 effectively mitigates lipid peroxidation caused by hypoxia, supporting the notion that ferroptosis contributes to neuronal loss insulted by hypoxia.

### Downregulation of CIRBP induced by hypoxia is crucial for nervous system development and iron homeostasis

**Fig. 4:**
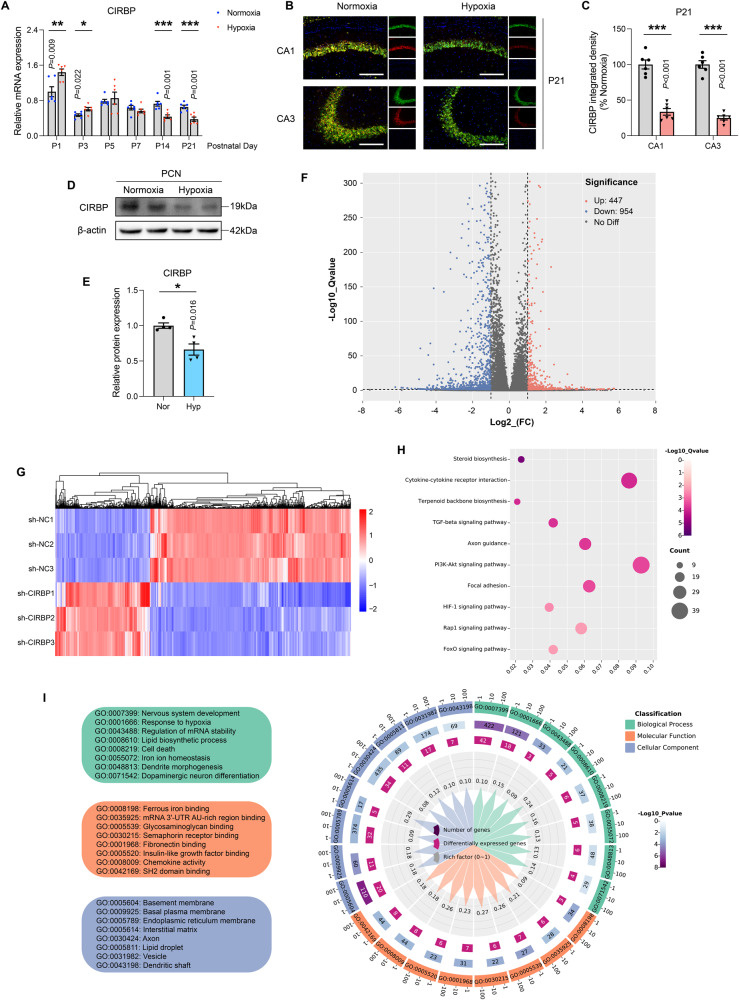
Hypoxia-induced CIRBP downregulation is essential for iron homeostasis and nervous system development. **A** The relative mRNA expression of CIRBP in hippocampus under normoxia or hypoxia on the designated date after birth, normalized to normoxia on P1 (*n* = 6). **B** The representative images of immunostaining (green for NeuN, red for CIRBP, blue for DAPI) in the CA1, CA3 regions of hippocampus. Scale bars is 200 μm. **C** Quantitative analysis of fluorescence intensity of CIRBP in hippocampal neurons. **D** Hypoxia-exposed primary neurons were homogenated and lysed and protein samples were prepared. Expression levels of CIRBP were tested by immunoblotting. **E** Intensity quantification was performed by Image J and normalized to β-actin (*n* = 4). **F** HT22 cells were transfected with lentivirus expressing shRNA against CIRBP (sh-CIRBP) or scrambled hairpin (sh-NC) followed by transcriptome RNA-sequencing. Volcano plot showed the differentially expressed genes. The −log_10_ *Qvalue* of each gene was plotted against the log_2_ *FC* of sh-CIRBP group to sh-NC group. Red dots (*FC >* 2, *Q <* 0.05) were considered as significantly upregulated genes, blue dots (*FC <* 0.5, *Q* < 0.05) indicated down-regulated genes. **G** Hierarchical cluster analysis and heat-map analysis to differential gene-level expression in sh-CIRBP cells. The dendrograms at the top of the heatmaps was used to depict the clustering (based on *z* scores) of the samples. Color bar at the right represented the color scale reflecting gene-level expression differences. **H** The top 10 most statistically significant pathways were demonstrated by KEGG pathway analysis. The color bar on the right represented the reflected -log_10_ *Q*value. Sizes of the circles represented the number of differential genes involved in the pathway. **I** GO enrichment analysis was utilized in biological process, molecular function, and cellular component terms. The colorful picture on the left represented the descriptions of different GO IDs. Data were showed as mean ± standard error of mean (SEM) of at least three independent experiments. Statistical analyses were carried out using a two-tailed unpaired Student’s *t* test for comparations between two groups. *P ≤ 0.05, **P ≤ 0.01, ***P ≤ 0.001. Source data are provided as a Supplemental Material file.

Numerous studies have demonstrated the neuroprotective properties of CIRBP [29, 30], although it remains unclear how these properties affect ferroptosis in neurons. Here, we discovered that CIRBP mRNA was upregulated in the early stages of exposure and considerably decreased in the late stages of continuous exposure in the model of hypoxic exposure during pregnancy (Fig. 4A). Additionally, the red fluorescence intensity of CIRBP (NeuN in green, DAPI in blue) decreased in accordance with the transcription level of CIRBP (Fig. 4B, C and SFig. 4A, B). Hippocampal CIRBP protein expression was downregulated as a result of hypoxia exposure in the hippocampus of P14 and P21 littermates as evident by western blot analysis (SFig. 4C, D). corroborating results were also obtained in the in vitro studies (Fig. 4D, E and SFig. 4E, F).

In order to further explore the biological role of CIRBP in cells and investigate whether CIRBP mediates in the progression of hypoxia-induced neuronal ferroptosis, we constructed lentivirus containing sh-CIRBP in HT22 cell lines to knock down CIRBP. Silencing efficiency was verified at the mRNA level (SFig. 6O). To explore differences in hypoxia or ferroptosis-related genes expression, we performed transcriptome sequencing in sh-CIRBP samples. Once the data were normalized, 1401 differentially expressed genes were identified, including 447 up‐regulated and 954 down‐regulated genes, as shown in the volcano map and heat map (Fig. 4F, G). In addition, KEGG pathway analysis revealed that axon guidance and the HIF-1 signaling pathway were significantly perturbed in the CIRBP knock-down cells (Fig. 4H), consistent with a key role for CIRBP in neuronal axon development and hypoxic stress signaling. Gene ontology (GO)-enrichment analysis (Fig. 4I) showed that nervous system development, regulation of mRNA stability, cell death, and iron ion homeostasis were significantly enriched in biological process terms, while ferrous iron binding and mRNA 3′-UTR AU-rich region binding were significantly enriched in molecular function terms. Taken together, these results indicate that attenuated expression of CIRBP may lead to neurological diseases and that the CIRBP binding element is likely located in regions corresponding to iron homeostasis-related genes.

### Overexpression of CIRBP attenuates hypoxia-induced cytotoxicity and lipid peroxidation

**Fig. 5:**
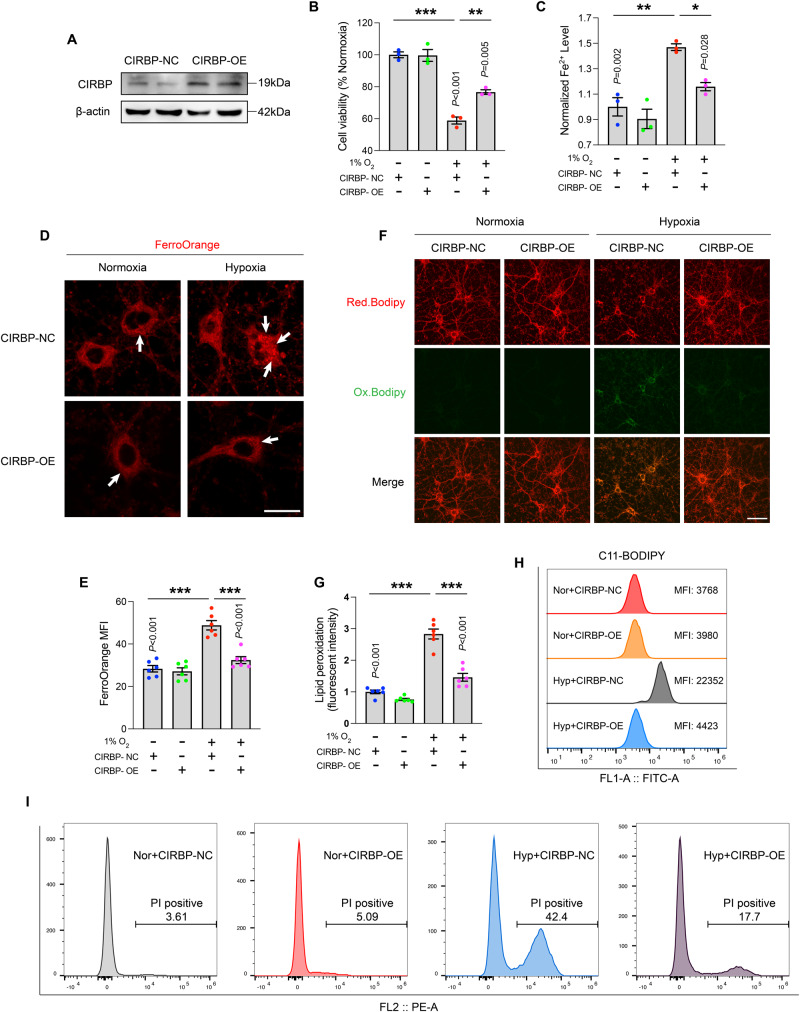
Hypoxia-induced cytotoxicity and lipid peroxidation are prevented by overexpressing CIRBP in vitro. **A** The overexpression efficiency of CIRBP validation by immunoblotting. **B** The primary neurons viability exposed by 1% O_2_ with or without CIRBP-OE administration (*n* = 3). **C** Quantitative analysis of ferrous ion content in primary neurons (*n* = 3). **D** The content of intracellular ferrous ions exposed to hypoxia or CIRBP-OE administration was detected by FerroOrange probe. Scale bar is 20 μm. **E** The iron levels are determined based on the intensities measured by Image J (*n* = 6). **F** The representative pictures of lipid peroxidation assay (red for reduction, green for oxidation) and **G** quantitative analysis for primary neurons under hypoxia or CIRBP-OE administration (*n* = 6). Scale bar is 50 μm. **H** The levels of C11-BODIPY in each group were determined by FACS analysis. **I** Cell death was measured by flow cytometry labeled PI staining. Data were showed as mean ± standard error of mean (SEM) of at least three independent experiments. Statistical analyses were carried out using two-way ANOVA and multiple *t* test. *P ≤ 0.05, **P ≤ 0.01, ***P ≤ 0.001. Source data are provided as a Supplemental Material file.

Given that hypoxia led to the downregulation of CIRBP, next, we aimed to assess the potential for CIRBP overexpression to mitigate the aberrant effects of hypoxia. A lentivirus plasmid (SFig. 5F) was generated to establish how CIRBP affects neuronal ferroptosis, with cells transduced with NC lentivirus serving as control (Fig. 5A). CCK-8 experiments demonstrated that exogenous CIRBP effectively reversed the 1% O_2_-induced loss in primary neuronal viability (Fig. 5B). Flow cytometry results demonstrated results corresponding to which CIRBP overexpression reduced the number of PI-positive cells in the hypoxic group (Fig. 5I). It was also observed (Fig. 5F, G) that the strong green fluorescence of Bodipy after oxidation induced by hypoxia was effectively attenuated by CIRBP overexpression. The FACS results also showed CIRBP overexpression notably reduced the amount of lipid peroxidation caused by hypoxia in HT22 cells (Fig. 5H). Confocal imaging revealed that ferrous ion content (red fluorescence) in primary neurons in the hypoxic group was attenuated upon CIRBP-OE (Fig. 5D, E). Analogous results were confirmed with the ferrous ion test kit (Fig. 5C). Taken together, the preceding in vitro results establish that CIRBP attenuated neuronal ferroptosis and lipid peroxidation induced by hypoxia.

### Conditional overexpression of CIRBP alleviates hypoxia-induced cognitive impairment in pups

**Fig. 6:**
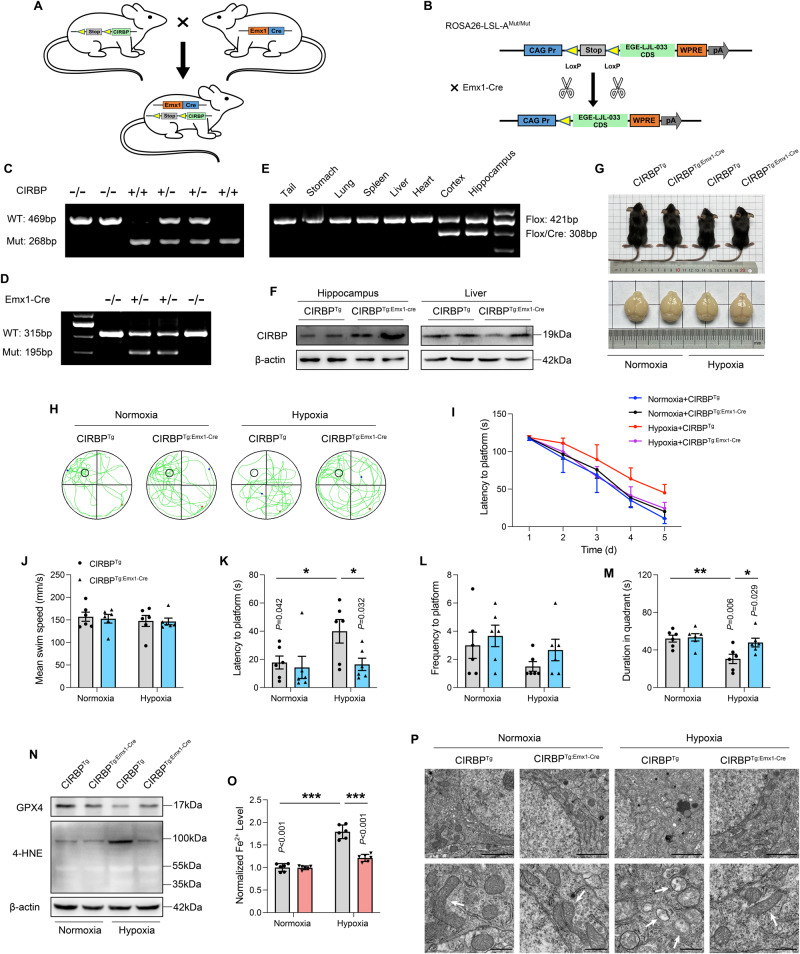
Conditional overexpression of CIRBP alleviates hypoxia-induced cognitive impairment in pups. **A** Cross-mating pattern CIRBP^Tg^ mice × Emx1-Cre mice. **B** The CIRBP gene was knock-in by Cre/loxP stop loxP recombination to generate mice with specific enhancement of CIRBP. **C**, **D** Genotyping PCR on genomic DNA from mouse tails of CIRBP^Tg^ mice and Emx1-Cre mice. **E** PCR analysis of tissue genomic DNA from a CIRBP^Tg: Emx1-Cre^ mouse. **F** The protein level of CIRBP in the primary neurons of hippocampus and liver tissues of CIRBP^Tg: Emx1-Cre^ mice and age-matched CIRBP^Tg^ littermates. β-actin was served as a loading control. **G** The representative images of CIRBP^Tg: Emx1-Cre^ mice with its whole brain and age-matched littermates (CIRBP^Tg^) exposed by hypoxia. **H** The representative swimming tracks of CIRBP^Tg: Emx1-Cre^ mice and age-matched littermates in the Morris water maze (*n* = 6). **I** The latency to the platform during the learning stages of CIRBP^Tg: Emx1-Cre^ mice and age-matched littermates under hypoxia conditions. **J** The mean swim speed, **K** the latency to a hidden platform, **L** the frequency to a hidden platform, as well as **M** the time in the target quadrant on the test day of CIRBP^Tg: Emx1-Cre^ mice and age-matched littermates in the Morris water maze (*n* = 6). **N** Protein samples of hippocampus were collected and ferroptosis-related proteins were detected by immunoblotting. Represented bands for GPX4, 4-HNE in the hippocampus. **O** The tissue iron content in the hippocampus of CIRBP^Tg: Emx1-Cre^ mice and age-matched littermates exposed by hypoxia, data were normalized to normoxia (*n* = 6). **P** Transmission electron microscopy pictures show conditional enhancement of CIRBP ameliorated shrunken mitochondria of hippocampal neurons under hypoxia conditions. Scale bars upper is 2 μm. Scale bars lower is 500 nm. Data were showed as mean ± standard error of mean (SEM) of at least three independent experiments. Statistical analyses were carried out using two-way ANOVA and multiple *t* test. *P ≤ 0.05, **P ≤ 0.01, ***P ≤ 0.001. Source data are provided as a Supplemental Material file.

To better characterize the role of CIRBP in cognitive dysfunction and brain iron metabolism, we generated CIRBP conditionally overexpressed homozygous mice (CIRBP^Tg: Emx1-Cre^) by crossing the CIRBP^Tg^ mice with Emx1-Cre mice [31]. The Cre enzyme was overexpressed in the neocortex and hippocampal pyramidal neurons (Fig. 6A, B), and the floxed littermates without Cre positive (CIRBP^Tg^) served as the control group. Genotype identification of CIRBP^Tg^ mice and Emx1-Cre mice is shown in Fig. 6C, D. In order to verify the efficiency of the Cre enzyme, we collected tissues (hippocampus, cortex, heart, liver, spleen, lung, stomach, and tail) of CIRBP^Tg: Emx1-Cre^ mice for genotype detection, and Cre enzyme only played a role in hippocampus and cortex (Fig. 6E). Western blot results also confirmed the overexpression of CIRBP in the hippocampus of CIRBP^Tg: Emx1-Cre^ mice, but no difference was ascertained in liver (Fig. 6F). To validate the efficiency of neuron-specific CIRBP overexpression, we examined CIRBP expression levels in neurons versus glial cells (SFig. 5A, B). As noted in Fig. 6G, CIRBP^Tg: Emx1-Cre^ mice showed comparable body and whole brain weights to their littermates floxed controls under normal conditions. However, CIRBP^Tg: Emx1-Cre^ mice showed greater body length, body weight, and brain weight (SFig. 5C–E) under hypoxia, demonstrating that neuron-specific enhancement of CIRBP can alleviate the abnormal development of offspring caused by hypoxic exposure.

Next, we performed behavioral assessment in CIRBP^Tg: Emx1-Cre^ mice and found no difference in basal locomotive ability between the CIRBP overexpressing mice and littermate controls (Fig. 6J and SFig. 5H). Compared with CIRBP^Tg^ mice in the hypoxic group, CIRBP^Tg: Emx1-Cre^ mice showed improved learning ability during the Morris water maze training sessions (Fig. 6I and STable 10), along with increased time and frequency to cross the platform and quadrant during the probe trial (Fig. 6H, K–M). The prolonged latency time to platform was alleviated by the overexpression of CIRBP. In the open field experiment, control mice (CIRBP^Tg^ mice) entered the central area less frequently, with shortened time and distance upon hypoxic exposure, and in CIRBP^Tg: Emx1-Cre^ mice this trend was alleviated (SFig. 5G, I–K). In addition, CIRBP-OE ameliorated the hypoxia-induced short-term learning impairment, characterized by a greater proportion of time spent on the novel objects (SFig. 5L, M). These results are consistent with the propensity of CIRBP upregulation to mitigate the perinatal hypoxia-induced memory impairments.

Since in vitro overexpression of CIRBP can effectively inhibit ferroptosis in neurons induced by hypoxia, we also addressed related indicators of ferroptosis in neuron-specific overexpressing CIRBP mice in vivo. As shown in Fig. 6N and SFig. 5N, O, CIRBP^Tg: Emx1-Cre^ mice showed comparable levels of GPX4 and 4-HNE as littermate controls under normoxic condition. CIRBP-OE enhanced GPX4 and attenuated 4-HNE expression under hypoxic condition. Furthermore, the abnormal iron accumulation inherent to mice undergoing hypoxic insult was partially reversed in the CIRBP overexpressing mice (Fig. 6O). Consistent with the amelioration of ferroptosis-related genes expression, smaller, ruptured mitochondria in hippocampus were partially rescued by CIRBP overexpression (Fig. 6P). Altogether, these results suggest that neuron-specific increased CIRBP levels can partially reduce susceptibility to hypoxia-induced ferroptosis.

### CIRBP prevents FTMT degradation to inhibit ferroptosis

**Fig. 7:**
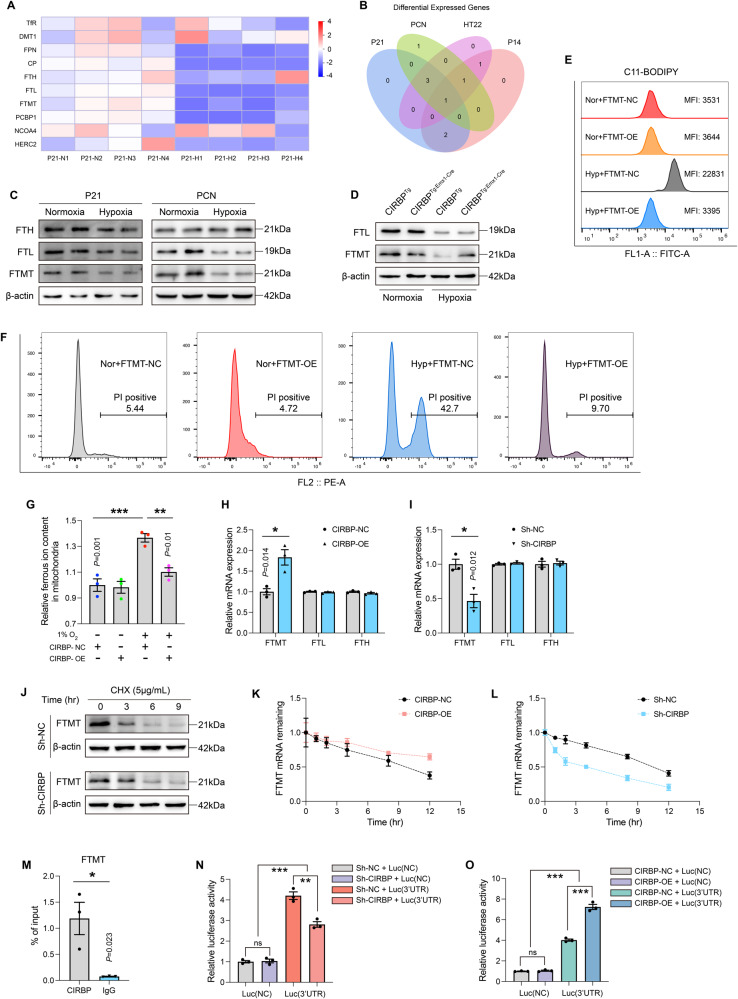
CIRBP delays the degradation of FTMT to inhibit ferroptosis. **A** Heat-map analysis to ferrous metabolism gene-level expression of P21 hippocampus under hypoxia exposure. **B** Venn diagram analysis of differential gene levels under hypoxic exposure in four models. **C** The expression level of ferritin-related proteins in the P21 hippocampus and the primary neurons under hypoxia conditions. **D** The representative western blot bands of FTL and FTMT in CIRBP^Tg: Emx1-Cre^ mice and age-matched littermates exposed by hypoxia. **E** The levels of C11-BODIPY in each group were determined by FACS analysis. **F** Cell death was measured by flow cytometry labeled PI staining. **G** The mitochondrial ferrous ions extracted from mitochondria in primary neurons for detection (*n* = 3). **H**, **I** The relative mRNA expression of ferritin-related genes in stably CIRBP-overexpression and CIRBP knockdown HT22 cells (*n* = 3). **J** CIRBP was detected by western blotting in CIRBP knockdown HT22 cells after treated with CHX (5 μg/mL). **K**, **L** FTMT mRNA decay line chart of stably CIRBP-overexpression and CIRBP knockdown HT22 cells after treated with actinomycin D (10 μg/mL). **M** RIP assay was performed with anti-CIRBP antibodies and control antibodies (IgG) in HT22 cells (*n* = 3). **N**, **O** Relative luciferase activity of reporter plasmids containing FTMT mRNA 3′UTR in stably CIRBP knockdown and CIRBP-overexpression HT22 cells (*n* = 3). Data were showed as mean ± standard error of mean (SEM) of at least three independent experiments. Statistical analyses were carried out using two-way ANOVA and multiple *t* test. *P ≤ 0.05, **P ≤ 0.01, ***P ≤ 0.001. Source data are provided as a Supplemental Material file.

As we ascertained abnormal iron accumulation in the hippocampus of hypoxic animals, and the crucial role of CIRBP in attenuating iron overload, next, we addressed specific mechanism of iron regulation by CIRBP. Specifically, we assessed the expression of genes related iron import (TfR, DMT1) [32, 33], iron export (FPN, CP) [34, 35], ferritin (ferritin heavy chain, ferritin light chain, FTMT, PCBP1) [36, 37], ferritinophagy (NCOA4, HERC2) [38, 39]. Heat map results showed that the ferritin pathway was significantly downregulated both in vivo and vitro in the hypoxic models (Fig. 7A and SFig. 6A-C). Furthermore, Venn diagram analysis revealed that FTMT was the only gene that was stably reduced (Fig. 7B). In addition, western blot results demonstrated a comparable level of ferritin heavy chain and decreased levels of ferritin light chain and FTMT in all hypoxic models (Fig. 7C and SFig. 6D-J). We next explored whether the CIRBP-mediated ferritin pathway, and observed that FTMT expression was reversed in the hippocampus of CIRBP^Tg: Emx1-Cre^ mice upon hypoxia, even though FTL protein expression was not regulated by CIRBP overexpression (Fig. 7D; SFig. 6K, L). Consistently, the accumulation of ferrous ions in mitochondria was also ameliorated by CIRBP (Fig. 7G). To further validate the role of FTMT we observed in all hypoxic models, we explored the effects of FTMT on lipid peroxidation and cell survival using a FTMT-overexpressing cell line in the hypoxia model. The efficiency of FTMT overexpression was verified by RT-PCR experiments (SFig. 6M). To our delight, enhanced FTMT expression effectively alleviated hypoxia-elevated lipid peroxidation (Fig. 7E) and significantly reduced the HT22 cell mortality (Fig. 7F).

To confirm a regulatory relationship between CIRBP and ferritin at the mRNA levels, we assessed the expression of FTH, FTL, and FTMT in CIRBP-OE and sh-CIRBP cells. RT-PCR was applied to verify overexpression or knockdown efficiency of CIRBP (SFig. 6N, O). The results showed that the expression of FTMT mRNA was significantly different between the two cell types, while other ferritin genes such as FTH and FTL were only slightly altered and statistically indistinguishable (Fig. 7H, I). Assuming that CIRBP-mediated FTMT levels via the protein degradation pathway, we treated sh-CIRBP cells with CHX at different time points, to inhibit protein synthesis. Western blot results revealed that sh-CIRBP did not change the half-life of FTMT protein (Fig. 7J). Considering the role of CIRBP as an RNA-binding protein and for ferrous ion binding, we determined FTMT mRNA stability in CIRBP-OE and sh-CIRBP cells by blocking mRNA synthesis with actinomycin D (a specific transcription inhibitor) at the designated time points. Interestingly, qPCR results showed that the half-life of FTMT mRNA was prolonged in CIRBP-OE cells and reduced in the sh-CIRBP groups (Fig. 7K, L and STable 11 and 12). To further fortify the above results, RIP assay was performed, showing a significant FTMT mRNA enrichment in the anti-CIRBP groups compared with anti-IgG (Fig. 7M). As the GO analysis described above has shown that mRNA 3’-UTR AU-rich region binding is enriched with CIRBP, we speculated that CIRBP promoted FTMT mRNA stability by directly binding to AU-rich region of 3’UTR. Thus, a luciferase reporter plasmid containing the FTMT mRNA 3’UTR was transfected into CIRBP-OE and sh-CIRBP cells, establishing that CIRBP-OE increased while sh-CIRBP repressed the luciferase activity (Fig. 7N, O). In summary, these results demonstrate that CIRBP directly binds to FTMT mRNA 3’UTR, increasing FTMT mRNA stability and thereby inhibiting ferroptosis.

## Discussion

High altitude introduces environmental stressors which are distinct from those encountered at sea level. It is estimated that over 14.4 million humans worldwide live at ≥ 3500 meters, while China has the greatest percentage of its population [40]. Based on findings reported in the Seventh National Census, approximately 443400 Han immigrants live in Tibet, with a significant number of pregnant women living residing in high altitude. Increased radiation, colder and drier climates, and hypobaric hypoxia in particular may cause difficulties in successful reproduction. Dolma noted that high altitude was associated with altered fetal growth and development via the study of the prospective population cohort [41]. In addition, it has been shown a reduction in neuronal number in the cortex, brain stem, and hippocampal CA1 regions of neonatal mice [42]. Hypoxia also contributes to aberrant synaptic shaping and function [43]. In our study, consistently, we found that perinatal hypoxia insult resulted in abnormal development and cognitive impairments in offspring, manifested by weight loss, decreased short-term memory and spatial learning deficits, as well as concomitant reduction in neuron counts.

Massive studies have shown that hypoxia-ischemia leads to neuronal apoptosis [44, 45]. Paradoxically, no neuronal apoptosis was found in our perinatal hypobaric hypoxia model, possibly due to the absence of the insult caused by ischemia. Ferroptosis is a novel non-apoptotic form of programmed cell death mediated by the overaccumulation of ferrous ions in cells [46]. Emerging studies have demonstrated a widespread relationship between ferroptosis and the pathophysiology of neurodegenerative illnesses, such as AD, PD, Huntington’s disease, and amyotrophic lateral sclerosis [47–50]. Recent studies have shown that mitochondrial complex III produces increased ROS levels in a hypoxic environment with 1% oxygen, damaging mitochondrial membrane and directly triggering lipid peroxidation, leading to ferroptosis [51, 52]. In this novel study, we identified relevant ferroptotic indices both in vitro and in vivo to determine whether ferroptosis contributes to neuronal deficits. The findings demonstrated that exposure to hypoxia facilitated intracellular ferrous ion accumulation, disturbances in the expression of genes involved in ferroptosis regulation and led to shrunken mitochondria. The effects of Lip-1 intraperitoneally on spatial learning, memory, and cognitive function were significantly improved in hypoxia-exposed littermates. Additionally, administration of ferroptosis inhibitor Fer-1 in vitro effectively alleviated both hypoxia-induced neuronal deficits and lipid peroxidation. These results demonstrate that ferroptosis was associated with perinatal hypobaric hypoxia-induced neuronal damage.

CIRBP belongs to a family of cold shock proteins that are activated in response to mild hypothermic stress [53, 54]. Besides moderate cold shock, CIRBP can also regulate the expression by self-transcriptional activation of alternative promoters under hypoxic condition [55]. CIRBP expression is upregulated during acute hypoxia, while persistent hypoxia inhibits its expression through an unknown mechanism. Previous studies have reported that hypoxia can inhibit the transcription of several genes by reducing the activity of ten eleven transformation enzyme (TET), leading to hypermethylation of the gene promoter region. CIRBP could also prompt CoQ10 biosynthesis, thus enhancing the antioxidant capacity of cells [16]. In our experiment, early upon hypoxic exposure, we found elevated CIRBP expression and no signs of coinciding ferroptosis. To the contrary, when hypoxic exposure persisted through P14 or P21, CIRBP showed a trend toward down-regulation, and ferroptosis occurred. Therefore, we hypothesize that neurons upregulate CIRBP expression as a protective anti-ferroptosis response to early hypoxic insult, with ensuing down-regulation in the latter stages of hypoxic exposure. To further understand the association between CIRBP and hypoxia-induced ferroptosis, we generated a mouse strain with selective enhancement of CIRBP in the neurons of the hippocampus and neocortex, and found that CIRBP^Tg: Emx1-Cre^ mice showed attenuated hypoxia-induced impairment in cognitive function and learning memory. In agreement with these findings, overexpression of CIRBP by lentiviral transfection ameliorated ferrous ion accumulation and lipid peroxidation upon hypoxia.

As an RNA binding protein, CIRBP is a crucial regulator which impacts the localization, translation, and stability of target genes [56–58]. By performing KEGG pathway and GO analysis on differentially expressed genes, we found that CIRBP played a key role in iron ion homeostasis and mRNA 3’-UTR AU-rich region binding. Since hypoxia caused excess iron accumulation, we evaluated four pathways of iron metabolism, including iron import, iron export, ferritin, and ferritinophagy. Heat map and Venn diagram results established that FTMT was the only gene stably reduced under hypoxic condition. We then used overexpression of FTMT lentivirus to validate its critical role in hypoxia-induced neuronal ferroptosis, and found that supplementation with FTMT significantly ameliorated neurotoxic injury caused by hypoxia. Furthermore, we discovered sh-CIRBP expression alone suppressed FTMT expression. Though CIRBP did not change the half-life of FTMT protein in CHX assays, mechanistically, we found that the CIRBP-OE groups had robust FTMT mRNA enrichment by directly binding to 3’-UTR AU-rich region in the mRNA, indicating that CIRBP promoted FTMT stabilization to alleviate ferrous ion accumulation.

Our experiments have several limitations. For example, ferroptosis inhibitors failed to completely reverse the neuronal loss induced by hypoxic insult, demonstrating the complexity of the mechanisms associated with hypoxia-induced neurotoxicity, and highlighting the possibility that other death mechanisms are concomitantly involved. Nonetheless, notably CIRBP did partially alleviate cellular lipid peroxidation and thus hypoxia-induced ferroptosis.

In conclusion, our findings provide evidence that pups subjected to hypoxia develop cognitive problems secondary to neuronal ferroptosis. Furthermore, our findings highlight the essential role of CIRBP as an RNA-binding protein in ferrous ion metabolism. Thus, targeting CIRBP modulation or inhibiting ferroptosis might offer a novel and promising therapeutic strategy to mitigate hypoxia-induced neurotoxicity.

### Reporting summary

Further information on research design is available in the Nature Research Reporting Summary linked to this article.

### Supplementary information

Supplementary Figure Legends

Supplementary Figure 1

Supplementary Figure 2

Supplementary Figure 3

Supplementary Figure 4

Supplementary Figure 5

Supplementary Figure 6

Supplementary Figure 7

Supplementary Tables

Reporting Summary

## Data Availability

All data generated or analyzed during this study are included in this published article and its supplementary information files.
