# Supplementary material for: Cold-inducible RNA binding protein alleviates iron overload-induced neural ferroptosis under perinatal hypoxia insult
Source: Cell Death Differ. 2024 Feb 22;31(4):524–39. doi: 10.1038/s41418-024-01265-x (PMC11043449; doi:10.1038/s41418-024-01265-x)
Supplement: Supplementary file 1 — Supplementary Figure Legends [file 41418_2024_1265_MOESM1_ESM.docx]

**SFigure legends**

**SFig. 1 Hypoxia accelerates cognitive abnormalities and neuronal deficits in littermates.**

**A** The representative hippocampus images of the P21 mice under normoxia or hypoxia conditions. **B** The hippopotamus weights of P21 mice in perinatal hypoxia insult and age-matched controls. **C** The representative trace of mice on P21 under hypoxia conditions in the open field test. **D** The total distance, **E** frequency to center, **F** duration in center and **G** central distance of mice between normoxia and hypoxia group (*n*$=$6). **H-J** The representative images of immunostaining (green for NeuN, blue for DAPI) in the CA1, CA3, DG regions of hippocampus on P5, P7 and P14 under hypoxia condition. Scale bars is 200 μm. **K-M** Counts of NeuN+ neurons in CA1, CA3 and DG regions of P5, P7 and P14 mice. **N** Intensity quantifications for apoptosis-related proteins, respectively (*n*$=$3). **O** Counts of TUNEL positive cells in CA1, CA3 and DG regions of P21 mice. Data were showed as mean$\pm$standard error of mean (SEM) of at least three independent experiments. Statistical analyses were carried out using a two-tailed unpaired Student’s *t* test for comparations between two groups. **P*$\leq$0.05, ***P*$\leq$0.01, ****P*$\leq$0.001. Source data are provided as a Supplemental Material file.

**SFig. 2 Hypoxia exposure exacerbates oxidative levels and mitochondrial damage in vivo.**

**A-C** Intensity quantifications for ferroptosis-related proteins, respectively (*n*$=$4). **D** The GSH/GSSG ratio in the hippocampus of mice with or without intraperitoneal Lip-1 (n = 8). **E** Protein samples of P5 and P7 hippocampus were collected and ferroptosis-related proteins were detected by immunoblotting. Represented bands for GPX4 and 4-HNE in hippocampus. **F, G** Intensity quantifications for ferroptosis-related proteins, respectively (*n*$=$4). **H** The GPx activity (mU/mg protein) in the hippocampus and cortex of P14 pups (*n*$=$6). **I** Transmission electron microscopy pictures show shrunken mitochondria of P14 hippocampal neurons under hypoxia conditions. Scale bars is 1 μm. **J** Frequency of mitochondrial area size around the nucleus demonstrated. The number of mitochondria is counted as 40, approximately. **K** The total distance, **L** frequency to center, **M** duration in center and **N** central distance of mice between normoxia and hypoxia group with intraperitoneal Lip-1 in the open field test (*n*$=$8). Data were showed as mean$\pm$standard error of mean (SEM) of at least three independent experiments. Statistical analyses were carried out using a two-tailed unpaired Student’s *t* test for comparations between two groups. **P*$\leq$0.05, ***P*$\leq$0.01, ****P*$\leq$0.001. Source data are provided as a Supplemental Material file.

**SFig. 3 Hypoxia exposure leads to ferroptosis and iron overload in HT22 cells.**

**A** HT22 cell line was exposed to 1% O_2_ for 6, 12, 24, and 48 hours. The cell viability was accessed by CCK8 assays (*n*$=$3). **B** The content of intracellular ferrous ions exposed to hypoxia for 48 hours was detected by FerroOrange probe. Scale bar is 20 μm. **C** The iron levels are determined based on the intensities measured by ImageJ (*n*$=$6). **D** The GSH content and **E** the GSH/GSSG ratio in the HT22 cells under hypoxia exposure. **F, G** The cell viability of HT22 and SH-SY5Ycells exposed by hypoxia with or without Fer-1 (1 μmol/L) administration (*n*$=$3). **H** The levels of C11-BODIPY in SH-SY5Y cells exposed by hypoxia with or without Fer-1 were determined by FACS analysis. **I** PI staining was utilized in HT22 cells viability assessment (red for PI, blue for DAPI), PI/DAPI dual-positive cells were considered to dead cells. Scale bar is 200 μm. **J** The representative pictures of lipid peroxidation assay for HT22 cells (red for reduction, green for oxidation). Scale bar is 50 μm. **K** Quantitative analysis of the number of double-positive cells as a proportion of total cells (*n*$=$6). **L** Quantitative lipid peroxidation analysis (the ratio of green fluorescence to red fluorescence) for HT22 cells under hypoxia or Fer-1 administration (*n*$=$6). Data were showed as mean$\pm$standard error of mean (SEM) of at least three independent experiments. Statistical analyses were carried out using two-way ANOVA and multiple *t* test. **P*$\leq$0.05, ***P*$\leq$0.01, ****P*$\leq$0.001. Source data are provided as a Supplemental Material file.

**SFig. 4 CIRBP is downregulated by hypoxia insult.**

**A** The representative images of immunostaining (green for NeuN, red for CIRBP, blue for DAPI) in the CA1, CA3 regions of P14 hippocampus. Scale bars is 200 μm. **B** Quantitative analysis of fluorescence intensity of CIRBP in hippocampal neurons. **C** The hippocampus of P14 and P21 were homogenated and lysed and protein samples were prepared. Expression levels of CIRBP were tested by immunoblotting. **D** Intensity quantification was performed by Image J and normalized to β-actin (*n*$=$4). **E** The expression levels of CIRBP protein in HT22 cells were tested and **F** intensity quantification was performed by Image J. Data were showed as mean$\pm$standard error of mean (SEM) of at least three independent experiments. Statistical analyses were carried out using two-way ANOVA and multiple *t* test. **P*$\leq$0.05, ***P*$\leq$0.01, ****P*$\leq$0.001. Source data are provided as a Supplemental Material file.

**SFig. 5 Conditional enhancement of CIRBP ameliorates hypoxia-induced cognitive abnormalities in pups.**

**A, B** CIRBP-specific overexpression in neurons and glial cells was validated using immunofluorescence, respectively. Scale bars is 500 μm. **C** The body length of CIRBP^Tg: Emx1-Cre^ mice and age-matched CIRBP^Tg^ littermates under hypoxia conditions was recorded. **D** The weight and **E** brain weight of CIRBP^Tg: Emx1-Cre^ mice and age-matched littermates with or without hypoxia insult. **F** Schematic diagram of lentiviral overexpression plasmid. **G** The representative trace of CIRBP^Tg: Emx1-Cre^ mice and age-matched littermates under hypoxia conditions in the open field test. **H** The total distance, **I** frequency to center, **J** duration in center and **K** central distance of CIRBP^Tg: Emx1-Cre^ mice and age-matched littermates between normoxia and hypoxia group (*n*$=$6). **L** The representative travel tracks of CIRBP^Tg: Emx1-Cre^ mice and age-matched littermates in the novel object recognition test (*n*$=$6). **M** The discrimination index for mice in the novel object recognition test (*n*$=$6). **N, O** Intensity quantifications for ferroptosis-related proteins, respectively (*n*$=$4). Data were showed as mean$\pm$standard error of mean (SEM) of at least three independent experiments. Statistical analyses were carried out using two-way ANOVA and multiple *t* test. **P*$\leq$0.05, ***P*$\leq$0.01, ****P*$\leq$0.001. Source data are provided as a Supplemental Material file.

**SFig. 6 Hypoxia exposure leads to down-regulation of ferritin protein in vivo and in vitro.**

**A-C** Heat-map analysis to ferrous metabolism gene-level expression of P14 hippocampus, HT22 cells and primary neurons under hypoxia exposure. **D-F** Intensity quantifications for ferritin-related proteins, respectively (*n*$=$4). **G** The samples of P14 hippocampus and HT22 were homogenated and lysed and protein samples were prepared. Expression levels of ferritin-related proteins were tested by immunoblotting. **H-J** Intensity quantification was performed by Image J and normalized to β-actin (*n*$=$4). **K, L** Intensity quantifications for FTL and FTMT were performed by Image J and normalized to β-actin. **M** The overexpression efficiency of FTMT lentiviral plasmids were verified by q-PCR. **N, O** The overexpression and knockout efficiency of CIRBP lentiviral plasmids were verified by q-PCR. Data were showed as mean$\pm$standard error of mean (SEM) of at least three independent experiments. Statistical analyses were carried out using a two-tailed unpaired Student’s *t* test for comparations between two groups. **P*$\leq$0.05, ***P*$\leq$0.01, ****P*$\leq$0.001. Source data are provided as a Supplemental Material file.

**SFig. 7 Original images of representative western blots in all the Figures.**
