# Supplementary figures and images for: Cold-inducible RNA binding protein alleviates iron overload-induced neural ferroptosis under perinatal hypoxia insult
Source: Cell Death Differ. 2024 Feb 22;31(4):524–39. doi: 10.1038/s41418-024-01265-x (PMC11043449; doi:10.1038/s41418-024-01265-x)

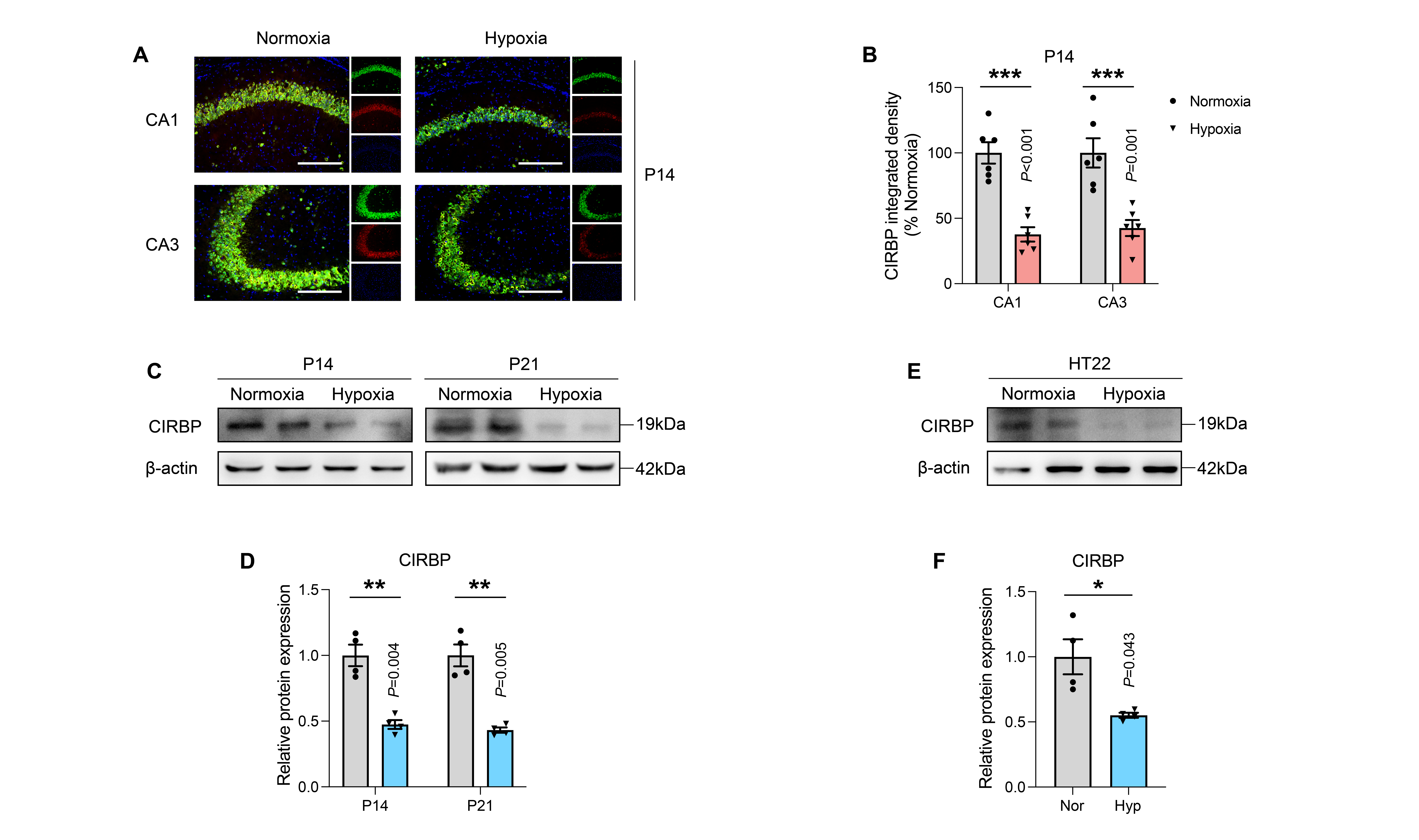

Supplement: Supplementary file 5 — Supplementary Figure 4 [file 41418_2024_1265_MOESM5_ESM.png]
