# Supplementary material for: Cold-inducible RNA binding protein alleviates iron overload-induced neural ferroptosis under perinatal hypoxia insult
Source: Cell Death Differ. 2024 Feb 22;31(4):524–39. doi: 10.1038/s41418-024-01265-x (PMC11043449; doi:10.1038/s41418-024-01265-x)
Supplement: Supplementary file 9 — Supplementary Tables [file 41418_2024_1265_MOESM9_ESM.docx]

Supplementary Tables

STable 1. Gene identification primer sequence

| Gene | Primer | Sequence (5’-3’) | Product size (bp) |
| --- | --- | --- | --- |
| CIRBP | ROSA-GT-F | AGTCGCTCTGAGTTGTTATCAG | WT: 469  Mut: 5172 |
|  | ROSA-GT-R | TGAGCATGTCTTTAATCTACCTCGATG |  |
|  | ROSA-GT-F | AGTCGCTCTGAGTTGTTATCAG | Mut: 268 |
|  | ROSA-PCR-R | AGTCCCTATTGGCGTTACTATGG |  |
| Emx1-Cre | Emx1-P1 | CAACGGGGAGGACATTGA | WT: 315  Mut: 195 |
|  | Emx1-P2 | CAAAGACAGAGACATGGAGAGC |  |
|  | Emx1-P3 | TCGATAAGCCAGGGGTTC |  |
| Flox-Cre | Cre-F | TGCTGGTTATTGTGCTGTCTCATCA | Flox: 421  Flox/Cre: 308 |
|  | Cre-R1 | CGAGAATGGTTATGTTCCTCCTCACT |  |
|  | Cre-R2 | TTTACCACCACCACTTCGGAGATCT |  |

STable 2. Primers used in qRT-PCR

| Target gene | Species | Sequence (5’-3’) |
| --- | --- | --- |
| Actin-F | mus | CTGTCCCTGTATGCCTCTG |
| Actin-R | mus | ATGTCACGCACGATTTCC |
| DMT1-F | mus | GAGCCCTTCACCACCTAC |
| DMT1-R | mus | AACGCCCAGAGTTTACGA |
| TfR-F | mus | GAGTGGCTACCTGGGCTAT |
| TfR-R | mus | TGTCTGTCTCCTCCGTTT |
| FPN-F | mus | GGGAGCATCAGCAATAAC |
| FPN-R | mus | CAGGCATGAATACGGAGA |
| CP-F | mus | CCCATTGATTGTTTGTCG |
| CP-R | mus | CAGGGTGTTCAGAGTATGTTTT |
| FTH-F | mus | AAGAAACCAGACCGTGAT |
| FTH-R | mus | TCAGTAGCCAGTTTGTGC |
| FTL-F | mus | TGAACCGCCTGGTCAACT |
| FTL-R | mus | CACGTCATCCCGATCAAA |
| FTMT-F | mus | AGCACATCAGCTCTGCACTG |
| FTMT-R | mus | AGGCCAGTAGGGGACCTAAA |
| PCBP1-F | mus | AGCATCATCGGGAAGAAAG |
| PCBP1-R | mus | AAAGATGGCATTGGTAGGC |
| NCOA4-F | mus | CGCCAGACCATCACCACA |
| NCOA4-R | mus | GTGCCACTGGATGCTGACTT |
| HERC2-F | mus | GCCGGACTGCTGTAGGTCTG |
| HERC2-R | mus | CAACCATTTGGAGTCAAGGCG |
| CIRBP-F | mus | CTCCGAAGTGGTGGTGGT |
| CIRBP-R | mus | GGCGTCCTTAGCGTCATC |

STable 3. Antibody information related to experimental procedures

| Antibody | Catalog number/manufacturer | Dilution |
| --- | --- | --- |
| NeuN | 66836-1-Ig, Proteintech | 1:1000 for WB |
| NeuN | ABN78, Millipore | 1:500 for IF |
| Gpx4 | ab125066, Abcam | 1:1000 for WB |
| 4-Hydroxynonenal | ab46545, Abcam | 1:1000 for WB |
| Slc7a11 | 12691S, CST | 1:1000 for WB |
| CIRBP | 10209-2-AP, Proteintech | 1:500 for WB |
| CIRBP | ab106230, Abcam | 1:500 for IF |
| Ferritin Heavy Chain | ab65080, Abcam | 1:500 for WB |
| Ferritin Light Chain | ab69090, Abcam | 1:1000 for WB |
| Mitochondrial Ferritin | ab66111, Abcam | 1:500 for WB |
| Bax | A0207, ABclonal | 1:1000 for WB |
| Bcl-2 | A0208, ABclonal | 1:1000 for WB |
| Caspase-3 | 9662, CST | 1:1000 for WB |
| actin | Ab8226, Abcam | 1:2000 for WB |

STable 4. CIRBP mRNA sequence

| ATGGCATCAGATGAAGGCAAGCTTTTCGTGGGAGGACTCAGCTTCGACACCAACGAGCAGGCGCTGGAGCAGGTCTTCTCCAAGTATGGGCAGATCTCCGAAGTGGTGGTGGTAAAGGACAGGGAGACCCAGCGATCCCGAGGCTTTGGGTTTGTCACCTTTGAAAATATCGATGACGCTAAGGACGCCATGATGGCTATGAATGGGAAGTCTGTGGACGGGCGGCAGATCAGAGTTGACCAGGCTGGCAAGTCTTCTGACAACCGGTCCCGAGGATACCGGGGTGGCTCTGCTGGAGGCCGGGGCTTTTTCCGTGGGGGACGAAGCCGGGGCCGAGGGTTCTCCAGAGGAGGAGGAGACCGGGGCTATGGAGGTGGCCGCTTTGAGTCCCGGAGTGGGGGTTATGGAGGCTCCAGAGACTACTATGCCAGCCGGAGTCAGGGTGGCAGCTATGGTTATCGGAGCTCGGGAGGGTCCTACAGAGACAGCTATGACAGTTATGGTAAGTCTAGCTCCAAGGATGCCATCTGA. |
| --- |

STable 5. FTMT 3’UTR sequence

| GACAAGAGGCTGCCTATCTGATACCCCAGTGGGTCCCAAGACCTGGATGGAGTTAACGATCTCCTGTTTCTTGCCCTTAAAACTTGACACCGTCTTTCCATTCTCCTCCTTCTTTGAACTGTTTTGCTAGTGTTTTGTAGAGAAAATATGCTAAGCCCTGTGCTCACTACGGCTTTCTACCAACACCAAAATACCAATTATTTCCCCAGCCTGAAAGATAGCCCAGAATACTATGGTATGACAGGGATGGGAGACAAAACACTTAAATTAGATACAAGAGTATGTTCTTTGAATATTATTAAGTGCTGTTCCTATCCATAAACCTGAACTGAAATTCCATGACTTCCAATTCAACCATCTGTACTGCCTTAAAAATCATATACAAAATGGTCGGTGAGAGAAATATATAATGTATAAATAATTGTAAGACAGAGAAGATATAGAGTACTTCAAAAATCACACATGCAAAATTTTGACAATTATTCTATTTTCCCAGTGTGTTCCAAGAAATGGCAGTGTATGGAGACAACCTAGGTATTGTTTTAAAGGATTCATGTCAAACTTGGTAAGAGCCACATCGGGTATCAGTGGTGGTGTTGAGCAGCTCAGTAGGCTCACATTTTAAGGGCTCAGAGAAGACTCTTAATTAAAACCACAGGAAAAAATATC |
| --- |

STable 6. Statistical package output for two-way ANOVA assessing the effect of exposure type and exposure time with interaction between the two factors on the weight of mice (Figure 1B)

| Source | Partial sum of squares | Degrees of freedom | Mean squares | F statistic | *p* value |
| --- | --- | --- | --- | --- | --- |
| Model | 472.819^a^ | 7 | 67.546 | 251.336 | 0.000 |
| Exposure type | 22.517 | 1 | 22.517 | 83.786 | 0.000 |
| Exposure time | 445.076 | 3 | 148.359 | 552.040 | 0.000 |
| Interaction | 2.899 | 3 | 0.966 | 3.595 | 0.020 |
| Residual | 12.900 | 48 | 0.269 |  |  |
| Total | 485.718 | 55 |  |  |  |

Note: a. R Squared = 0.973 (Adjusted R Squared = 0.970)

STable 7. Statistical package output for two-way ANOVA assessing the effect of exposure type and exposure time with interaction between the two factors on the brain weight of mice (Figure 1C)

| Source | Partial sum of squares | Degrees of freedom | Mean squares | F statistic | *p* value |
| --- | --- | --- | --- | --- | --- |
| Model | 0.339^a^ | 7 | 0.048 | 255.609 | 0.000 |
| Exposure type | 0.016 | 1 | 0.016 | 85.839 | 0.000 |
| Exposure time | 0.322 | 3 | 0.107 | 567.762 | 0.000 |
| Interaction | 0.000 | 3 | 9.656E-5 | 0.510 | 0.677 |
| Residual | 0.009 | 48 | 0.000 |  |  |
| Total | 0.348 | 55 |  |  |  |

Note: a. R Squared = 0.974 (Adjusted R Squared = 0.970)

STable 8. Statistical package output for two-way ANOVA assessing the effect of exposure type and exposure time with interaction between the two factors on latency (Figure 1E)

| Source | Partial sum of squares | Degrees of freedom | Mean squares | F statistic | *p* value |
| --- | --- | --- | --- | --- | --- |
| Model | 46675.330^a^ | 9 | 5186.148 | 29.802 | 0.000 |
| Exposure type | 24821.716 | 1 | 24821.716 | 142.635 | 0.000 |
| Exposure time | 18195.696 | 4 | 4548.924 | 26.140 | 0.000 |
| Interaction | 3657.919 | 4 | 914.480 | 5.255 | 0.001 |
| Residual | 8701.122 | 50 | 174.022 |  |  |
| Total | 55376.452 | 59 |  |  |  |

Note: a. R Squared = 0.843 (Adjusted R Squared = 0.815)

STable 9. Statistical package output for two-way ANOVA assessing the effect of exposure type and exposure time with interaction between the two factors on latency (Figure 2I)

| Source | Partial sum of squares | Degrees of freedom | Mean squares | F statistic | *p* value |
| --- | --- | --- | --- | --- | --- |
| Model | 260213.94^a^ | 19 | 13695.470 | 78.198 | 0.000 |
| Exposure type | 4163.066 | 3 | 1387.689 | 7.923 | 0.000 |
| Exposure time | 254047.649 | 4 | 63511.912 | 362.638 | 0.000 |
| Interaction | 2003.224 | 12 | 166.935 | 0.953 | 0.497 |
| Residual | 24519.426 | 140 | 175.139 |  |  |
| Total | 284733.365 | 159 |  |  |  |

Note: a. R Squared = 0.914 (Adjusted R Squared = 0.902)

STable 10. Statistical package output for two-way ANOVA assessing the effect of exposure type and exposure time with interaction between the two factors on latency (Figure 6I)

| Source | Partial sum of squares | Degrees of freedom | Mean squares | F statistic | *p* value |
| --- | --- | --- | --- | --- | --- |
| Model | 149796.35^a^ | 19 | 7884.018 | 57.045 | 0.000 |
| Exposure type | 7361.555 | 3 | 2453.852 | 17.755 | 0.000 |
| Exposure time | 139865.197 | 4 | 34966.299 | 252.997 | 0.000 |
| Interaction | 2569.597 | 12 | 214.133 | 1.549 | 0.119 |
| Residual | 13820.811 | 100 | 138.208 |  |  |
| Total | 163617.160 | 119 |  |  |  |

Note: a. R Squared = 0.916 (Adjusted R Squared = 0.899)

STable 11. Statistical package output for two-way ANOVA assessing the effect of CIRBP-overexpression and time with interaction between the two factors on remaining FTMT mRNA (Figure 7K)

| Source | Partial sum of squares | Degrees of freedom | Mean squares | F statistic | *p* value |
| --- | --- | --- | --- | --- | --- |
| Model | 1.144^a^ | 11 | 0.104 | 12.229 | 0.000 |
| CIRBP-OE | 0.076 | 1 | 0.076 | 8.908 | 0.006 |
| Time | 0.994 | 5 | 0.199 | 23.376 | 0.000 |
| Interaction | 0.074 | 5 | 0.015 | 1.746 | 0.163 |
| Residual | 0.204 | 24 | 0.009 |  |  |
| Total | 1.348 | 35 |  |  |  |

Note: a. R Squared = 0.849 (Adjusted R Squared = 0.779)

STable 12. Statistical package output for two-way ANOVA assessing the effect of CIRBP-knockdown and time with interaction between the two factors on remaining FTMT mRNA (Figure 7L)

| Source | Partial sum of squares | Degrees of freedom | Mean squares | F statistic | *p* value |
| --- | --- | --- | --- | --- | --- |
| Model | 2.376^a^ | 11 | 0.216 | 136.590 | 0.000 |
| sh-CIRBP | 0.439 | 1 | 0.439 | 277.442 | 0.000 |
| Time | 1.823 | 5 | 0.365 | 230.511 | 0.000 |
| Interaction | 0.115 | 5 | 0.023 | 14.497 | 0.000 |
| Residual | 0.038 | 24 | 0.002 |  |  |
| Total | 2.414 | 35 |  |  |  |

Note: a. R Squared = 0.984 (Adjusted R Squared = 0.977)
